# MATTE: a pipeline of transcriptome module alignment for anti-noise phenotype-gene-related analysis

**DOI:** 10.1093/bib/bbad207

**Published:** 2023-06-03

**Authors:** Guoxin Cai, Wenyi Zhao, Zhan Zhou, Xun Gu

**Affiliations:** Innovation Institute for Artificial Intelligence in Medicine and Zhejiang Provincial Key Laboratory of Anti-Cancer Drug Research, College of Pharmaceutical Sciences, Zhejiang University, Hangzhou 310058, China; Innovation Institute for Artificial Intelligence in Medicine and Zhejiang Provincial Key Laboratory of Anti-Cancer Drug Research, College of Pharmaceutical Sciences, Zhejiang University, Hangzhou 310058, China; Innovation Institute for Artificial Intelligence in Medicine and Zhejiang Provincial Key Laboratory of Anti-Cancer Drug Research, College of Pharmaceutical Sciences, Zhejiang University, Hangzhou 310058, China; The Fourth Affiliated Hospital, Zhejiang University School of Medicine, Yiwu, 322000, China; Department of Genetics, Development and Cell Biology, Iowa State University, Ames, IA 50011, USA

**Keywords:** transcriptomics, clustering, comparative analysis, module alignment, phenotype

## Abstract

A phenotype may be associated with multiple genes that interact with each other in the form of a gene module or network. How to identify these relationships is one important aspect of comparative transcriptomics. However, it is still a challenge to align gene modules associated with different phenotypes. Although several studies attempted to address this issue in different aspects, a general framework is still needed. In this study, we introduce Module Alignment of TranscripTomE (MATTE), a novel approach to analyze transcriptomics data and identify differences in a modular manner. MATTE assumes that gene interactions modulate a phenotype and models phenotype differences as gene location changes. Specifically, we first represented genes by a relative differential expression to reduce the influence of noise in omics data. Meanwhile, clustering and aligning are combined to depict gene differences in a modular way robustly. The results show that MATTE outperformed state-of-the-art methods in identifying differentially expressed genes under noise in gene expression. In particular, MATTE could also deal with single-cell ribonucleic acid-seq data to extract the best cell-type marker genes compared to other methods. Additionally, we demonstrate how MATTE supports the discovery of biologically significant genes and modules, and facilitates downstream analyses to gain insight into breast cancer. The source code of MATTE and case analysis are available at https://github.com/zjupgx/MATTE.

## INTRODUCTION

One of the significant objectives of multi-omics analysis, including transcriptomics, is to identify phenotype-associated genes or recognize related mechanisms. Comparative transcriptomics has a history of approaching the issue from the standpoint of a single gene, known as the differentially expressed (DE) gene. The most widely used techniques, including edgeR and DESeq2 [1–3], use sophisticated normalization to remove non-biological differences from the samples before identifying the differential expression of genes using linear statistical models. However, because of noise and complexity in transcriptomics data, the conventional technique could cause unexpected false positives [4, 5] and an inability to contain phenotypic casualties [6].

Interactome studies [7–9] have demonstrated that gene relationships are also important in describing the differences between phenotypes, which are not taken into account by DE approaches. As a result, researchers’ attention has gradually shifted from analysis from the perspective of a single gene to a module or network that can consider more complex relationships [10]. Co-expression is the most frequently discussed gene–gene interaction, and numerous studies have looked for significant gene correlations in a given condition, known as differentially co-expressed (DC) genes. For example, weighted correlation network analysis (WGCNA) [11] is a typical method to identify complex modules and networks from transcriptomics data. Module-based methods [12–14] have shown their ability to identify robust disease markers.

To identify gene modules that correlate with the change corresponding to a different phenotype, it is critical to resolve the issue of repeatability or preservation of modules, or more specifically, whether the module will arise again in samples from various phenotypes. Differentiated modules between normal and disorder samples are usually used to identify pathogenic factors in disease studies, while preserved modules usually identify conserved and essential functions in evolution studies [15–19]. However, there are several difficulties in identifying phenotype-related gene modules. First, noise is a critical confounder in the analysis of transcriptomics data, particularly when data are gathered from various batches or data sources. Second, complex patterns between modules may manifest when aligning. It is expected that a method will take note of these pattern differences. As the application scenarios differ, the results must be able to be interpreted and analyzed in both the preservation and differentiation modules. Therefore, a general analysis framework is urgently needed.

Many recent studies have focused on how to map modules built from different phenotype samples for the challenges mentioned previously, which raises the following new problem: how to define a suitable metric for deciding whether a module is preserved. As summarized by Langfelderin *et al.* [20], it can be roughly divided into two kinds of methods: network-based and cross-tabulation-based. The distinction between the two types of methods is based on whether the difference is found before or after clustering. Cross-tabulation-based methods accept the module assignments from two conditions as input and call for independent module identification. Accordingly, labels from two conditions are represented in rows and columns of a table. To establish the significance of module independence as determined by the overlaps of two labels, a statistical test is also performed. Although cross-tabulation methods seem logical, they overlook the complex pattern alterations and instead focus on checking if input modules are preserved [21]. Network-based methods build a network with genes as nodes and differences between two phenotypes as edges [22, 23]. Gene relationships in differential networks are informational, allowing for investigating intricate module patterns. However, network-based methods can only reveal significant modules when they are differentiated.

Motivated by the natural language process, in which a word in a sentence can be represented by its context, we hypothesize that gene expression should be considered in a global context. For the challenges mentioned above, a Module Alignment of TranscripTomE (MATTE) pipeline is constructed in this study to cluster and perform module alignment. To assess expression differences under the global expression atlas, MATTE uses relative differential expression (RDE) to define how differently a gene is expressed from others in each phenotype. A similar strategy can be applied to co-expression, defined as relative differential co-expression (RDC). MATTE has a strong anti-noise ability to detect both DE and DC genes due to the consideration of the relative difference in comparison to other differential clustering methods. Additionally, cross-tabulation and clustering techniques are combined to reveal complex module patterns, making differences between phenotypes more understandable and comprehensible. We further used MATTE to identify the genes and modules that truly distinguish between two or more phenotypes through three experiments, including simulations, high-noise single-cell transcriptomes and bulk ribonucleic acid (RNA)-seq data of breast cancer. The results demonstrate the advantages of MATTE, including stability, robustness and explainability.

## MATERIALS AND METHODS

### The framework of MATTE

**Figure 1:**
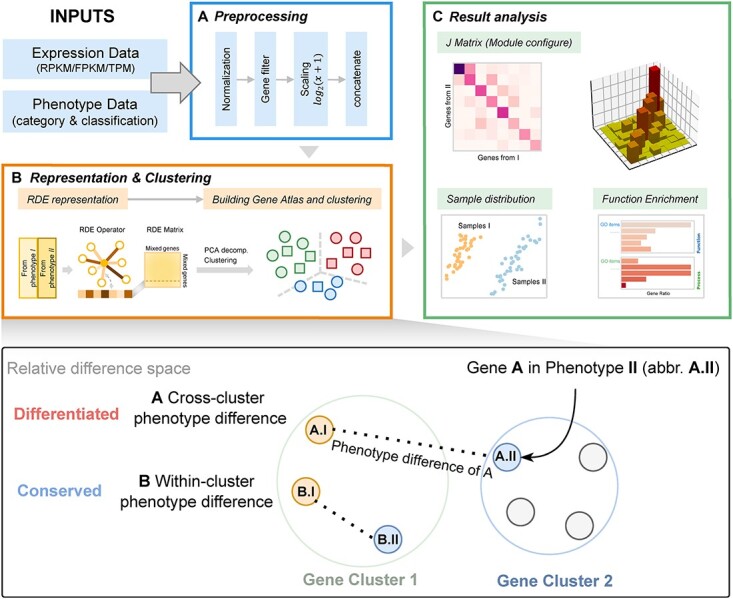
The analysis framework (top) and intuition (bottom) of MATTE. MATTE takes expression data and phenotype data as inputs. (**A**): Preprocessing: including normalization, filtering, scaling and finally concatenate data. (**B**): representation and clustering: using relative difference to characterize genes, decomposition for easy calculations and clustering genes from different phenotypes. Within-cluster difference is considered as conserved and vice versa. (**C**): Result analysis: relocate genes by their two labels (MC), and analysis specific gene set for function enrichment or sample presentation.

Most comparative transcriptome research used a strategy that separates clustering on various phenotypes and subsequent module alignment [21, 24]. In contrast, MATTE first constructs a discriminative representation of genes, emphasizing differences between phenotypes and then simultaneously clusters and aligns modules via cross-tabulation (Figure 1). Specifically, it consists of the following steps: (i) Preprocessing of transcriptomics data. (ii) Representing genes by relative difference and clustering. (iii) Align modules by cross-tabulation and analysis. The core idea of MATTE is to construct a special space in which each gene from each phenotype has a location. By comparing the difference in gene location between the two phenotypes, we can determine whether the gene has an apparent difference between the two phenotypes. This comparison cannot be carried out in the original data space because of the sample size disparity. This representation is consistent with the idea that the distance from three points in a two-dimensional plane can determine the position of an issue. Similarly, in nature language processing (NLP), words are expressed in the context of a phrase. A new problem arises in determining a differential change by distances in this space. We performed clustering to define the change in the location of genes. Compared to setting a threshold of distance, clustering is thought not to overfit and has an anti-noise ability. The results in the form of gene sets can make it easy to interpret the biological function difference between phenotypes. Specifically, genes from different phenotypes are considered independent and clustered together, further distinguished by analyzing changes in the labels of the homogenous gene in different phenotypes. After clustering, each gene has two labels corresponding to two phenotypes. All genes were further divided by the labels into subclusters containing the same two labels. If two labels of a gene corresponding to two phenotypes are the same, the gene is considered not to contribute to the divergences of two phenotypes. We constructed a matrix showing module overlaps for a more detailed analysis, drawing on the cross-tabulation method, where each unit is defined as module configuration (MC). It can be concluded that the differences in each MC lead to phenotypic differences.

### Notation

We use small cases to represent a vector, i.e. $x$ and large cases to represent a matrix, i.e. $X$. $\left\langle x\right\rangle =1/m\sum_i^m{x}_i$ and $\sigma (x)$ represent mean value and standard deviation of a vector $x$, respectively. $\left[X,Y\right]$ represents the concatenate of two matrices $X$ and $Y$. $pi$ and $gi$ represents different phenotypes and genes. $\left\{ gi; condition\right\}$ is a gene set that meets the condition.

### Data preprocessing

The data preprocessing includes three steps as follows: (i) Normalization: In bulk RNA sequencing data, fragments per kilobase of exon model per million mapped fragments or reads per kilobase of exon per million reads mapped (RPKM) in the data are converted to transcripts per kilobase million; (ii) Gene filtering: Delete genes with low expression. In processing breast cancer (BRCA) data from The Cancer Genome Atlas (TCGA) program, gene expression lower than one in over 90% of samples will be dropped. The threshold is set according to number of retained genes and their importance; (iii) Scaling: log transform was used to reduce the influence of genes with extremely high or low expression. All datasets used in this study and their source are listed in the Data availability section. As the datasets come from variable sources with variable formats and the preprocessing may differ, we provide Figure S1 to clarify the processing steps of each dataset.

### Gene embedding

MATTE aspires to create a space in which each gene ${g}_i$ from different phenotypes $p1$ and $p2$ is treated as an individual, i.e. ${g}_i^{p1}$ and ${g}_i^{p2}$. This allows for the observation of changes in the localization of genes from different phenotypes, reflecting the role of genes in phenotypic diversity. To create such a space, an identical gene description system and an anti-noise strategy that addresses the potential noise interference of data from multiple sources are needed. For transcriptomics data $X\in{\mathbb{R}}^{ng\times ns}$ with $ng$ genes and $ns$ samples, the difference between each gene and other genes is what we refer to as the RDE:


(1) \begin{equation*} RD{E}_{ab}=\left|\left\langle{X}_a\right\rangle -\left\langle{X}_b\right\rangle \right| \end{equation*}

where $a,b\in \left\{{g}_i^p;p\in \left\{p1,p2\right\}\right\}$. By doing so, the noise can be eliminated as a whole (Supplementary Methods), and a single gene receives a relative difference in global information. Such relative differences can be used with a variety of statistics, including Pearson correlation coefficients (PCCs) to identify co-expression differences and mean statistics to gage the degree of differential expression. In the condition of DC analysis, a normalized spectral embedding $L={D}^{-1/2}\left(D-A\right){D}^{-1/2}$ is performed based on the graph constructed from the PCC, followed by concatenation to obtain $RDC=\left[{L}^{p1},{L}^{p2}\right]$.

To facilitate the follow-up calculation, we reduce the dimension of the corresponding data by the most common principal component analysis method (PCA). By transforming and clustering relative differences, each gene will assign two labels ${l}_i^{p1}$ and ${l}_i^{p2}$ corresponding to two phenotypes.

### Construct and rank MCs

In this study, the absolute value distance of the PCC is used to cluster the gene embeddings with K-Means. Genes can be relocated to MCs using two labels that specify the row and column index (J matrix).


(2) \begin{equation*} {M}_{n,m}=\left\{{g}_i;{l}_i^{p1}=n,{l}_i^{p2}=m\right\} \end{equation*}

By doing so, modules can be robustly constructed for particular phenotypes and directly aligned. MCs with the same two labels in the diagonal of the J matrix are thought not to contribute to phenotype difference ($n=m$, conserved), whereas non-diagonal MCs do ($n\ne m$, differentiated). To evaluate contribution of a particular MC, the signal-to-noise ratio (SNR) of the module eigengene (ME) is calculated. Referring to previous studies [11, 25, 26], $M{E}_{n,m}= PC{A}^{(1)}\left({Y}_i\right),{g}_i\in{M}_{n,m}$ is the first principal component of data $Y$, which is the expression of genes in the MC ${X}_i$ when considering DE, and the inter-individual correlation (IIC) [25] for most variant gene pairs $k=\left\{k1,k2\right\}$ when considering DC (Supplement Methods).


(3) \begin{equation*} II{C}_k=\left({x}_{k1}-\left\langle{X}_{k1}\right\rangle \right)\left({x}_{k2}-\left\langle{X}_{k2}\right\rangle \right)/\left(\sigma \left({X}_{k1}\right)\times \sigma \left({X}_{k2}\right)\right) \end{equation*}

Based on the MATTE framework, we also designed a module-based gene rank method. The core idea is to represent the score of genes in the module by the total score of the module. This study uses the SNR to represent the significance of the MC, where samples are represented by the ME of the module. The absolute subtraction of the ME is treated as signal and the sum of the standard deviation is treated as noise. When analyzing multiple phenotypes, scores of phenotype pairs are added.


(4) \begin{equation*} SN{R}_M^{pi, pj}=\frac{\left|\left\langle{ME}_M^A\right\rangle -\left\langle{ME}_M^B\right\rangle \right|}{\sigma \left(M{E}_M^A\right)+\sigma \left(M{E}_M^B\right)} \end{equation*}

(5) \begin{equation*} SN{R}_g=\sum_{pi\in \mathbf{p}, pj\in \mathbf{p}, pi\ne pj} SN{R}_M^{pi, pj},g\in M \end{equation*}

where $A=\left\{{s}_1^i,{s}_2^i,\dots, {s}_n^i;p\left({s}_n^i\right)={p}_i\right\}$ is a set that contains all samples of one phenotype, $B$ is another set that presents the other phenotype.

### Simulation study

#### Data generation

We consider gene expression patterns as a mixture of two modes: DE and DC. The simulation is based on the multivariate normal distribution $X\sim \mathcal{N}\left(\mu, C\right)$, where $\mu$ is the mean of expression and $C$ is covariance matrix. There are nine mode combinations, as each mode has three levels: strong, weak and none.

It is known that the covariance matrix is the result of the PCC and standard deviation of two variables. Therefore, the covariance matrix can be obtained by generating the PCC matrix. In this study, it is considered that ${\sigma}_X={\sigma}_Y=1$. The properties of PCC can be summarized as follows: (i) it is a positive semidefinite matrix, and (ii) its diagonal elements are ones. The covariance matrix $C$ can be obtained by $C=B{B}^T$, where $B=\sqrt{\frac{D_{ij}}{\sum_{j=1}^{j=n}{D}_{ij}}}$ and $${D}_{ij}=\left\{\begin{array}{c}0,{e}_{ij}\ge \lambda \\{}1,{e}_{ij}<\lambda \end{array}\right.,{e}_{ij}\sim U\left(0,1\right)$$. It can be easily proven that $C$ is a positive semidefinite matrix whose diagonal element ${C}_{ii}=\sum{B}_{ij}^2=1$ and expectation $\mathbb{E}\left({A}_{ij}\right)=\lambda, i\ne j$, where $\lambda$ controls the correlation coefficients.

#### Noise simulation

For random noise, it is considered that the noise conforms to the normal distribution ${X}^{\prime }=X+\varepsilon$, where $\varepsilon \sim \mathcal{N}\left(0,I\right)$ and $I$ represents the variance of the normal distribution of the noise. A different $I$ value is taken to simulate the noise intensity. For batch-effects-like noise, we randomly select a certain proportion of genes ${G}_b.$ for the batch-effects-like noise and add a fixed amount of expression to them $${x}^{\prime }=x+\lambda \times \mathbb{B}(x)$$, where $$\mathbb{B}\left({X}_i\right)=\left\{\begin{array}{c}0,i\notin{G}_b\\{}1,i\in{G}_b\end{array}\right.$$

### Evaluation metrics

The performance of MATTE is assessed using three frequently used metrics for three different prediction task types. The AUC of the receiver operating characteristic curve (ROC) [27] is used for binary classification prediction. For multi-class prediction, F1 score [28] instead. The adjusted Rand index (ARI) [29] is used as the indicator to compare clustering labels with ground truth. For all three metrics, a higher score indicates a better performance. These criterions are implemented by scikit-learn package [30].

## RESULTS

### Simulations show the accuracy and robustness of the relative difference analysis of MATTE

In this section, we aim to demonstrate that relative difference is an accurate and robust metric to evaluate gene expression differences, especially under noise. We designed several simulation experiments and compared MATTE with two kinds of methods (See Supplementary Methods for details). In addition to methods that assign a score to each gene, some methods [31–33] seek to identify changes in a gene pair connection. In subsequent experiments, the sum of the distances between a particular gene and other genes, as well as RDE and RDC, were regarded as the gene score. We also considered combining RDE and RDC as a relative difference mixture (RDM) score to see if the two could be used together. The data containing differential expression patterns were generated under multivariate normal distribution, with nine differential expression patterns combining three DE or DC levels: strong, weak and none. In the simulation, DE genes are shown as the deviation of the mean value of genes in two conditions. DC genes are presented as a difference in connectivity to other genes in a differential co-expression network, where the PCC builds the graph. All DE and DC genes are labeled positive and others are labeled negative. Here, two metrics are used to evaluate methods to find DE genes. One is the area under curve (AUC) of the ROC curve, which assesses how well the score or rank of genes is consistent with the ground truth. The other is the ARI, which tests the similarity between the two clustering results.

**Figure 2:**
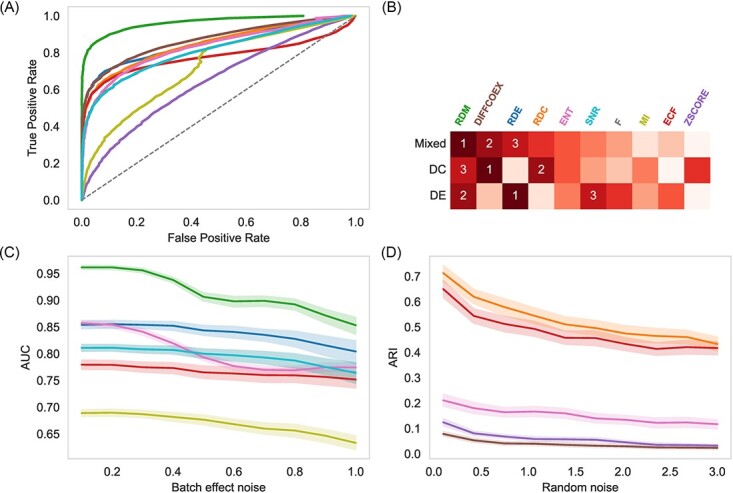
The performance of MATTE comparing with two kinds of methods in simulation experiments. (**A**): ROC curves of methods in simulations with mixing DE and DC pattern. (**B**): Rank heatmap of methods in three expression pattern simulations. The number in the heatmap represents the evaluation rank of the method. (**C**): AUC value of ROC when adding batch-effect like noise. (**D**): ARI of clustering results of before and after adding random noise. Line color indicates the method used, which corresponds to the color of x-axis labels in (B). Abbreviations and details of each method are provided in supplementary methods.

We begin by generating data with the mixed differential expression pattern described above and then evaluating methods by classifying DE genes. As shown in Figure 2A and B, RDE and RDC achieved the top two AUCs in DE and DC, respectively. By fusing the two indexes as RDM, DE and DC in complex expression mode can be identified (Supplementary Figure S2). For further analysis and comparison, we then calculate the accuracy of each differential pattern group at a threshold, making the false positive ratio to be 0.05. As shown in Supplementary Figure S3, RDM combines the advantages of both RDE and RDC, thus showing a better accuracy in gene groups with weak or no patterns. We also find that methods may be influenced by a complex expression pattern, as DC patterns can make some DE methods’ accuracy up or down. In general, combining DE and DC can obtain a more accurate result. The Expected Condition F-statistic (ECF) [31] claims its ability to incorporate location and correlation of expression, but only shows its weak ability to detect DC genes when there is no DE signal. Another method that shows comprehensive ability is entropy (ENT) but obtains a limited result at both DE and DC (Supplementary Figure S2a). In summary, the relative difference shows its advantages in determining DE genes with complex expression patterns.

To test the anti-noise ability of MATTE, we examined two kinds of noise based on the above experiments, batch-effect-like and random noise, affecting DE and DC signals respectively. First, we simulated the condition that the batch effect remains in part of the data. A value is randomly assigned to 20% of the genes in the generated data and the AUC is calculated as previously done. As shown in Figure 2C, MATTE (RDE and RDM) retains the highest AUC as the noise increases. In the random noise test, the result stability of downstream tasks is used to evaluate the robustness. We randomly selected 100 genes from real data (100 times, each time the genes were re-extracted) for clustering, and the consistency of the results with and without noise was evaluated. Compared with the traditional method of calculating the PCC from each edge, the correlation is easily affected by noise, while the RDC is more robust (Figure 2D). Unlike previous studies, RDC uses a relative view to see changes in gene correlations; thus, background changes in all pairs of genes would not affect the score of a gene pair.

### MATTE can identify marker genes in single-cell RNA-seq data

**Figure 3:**
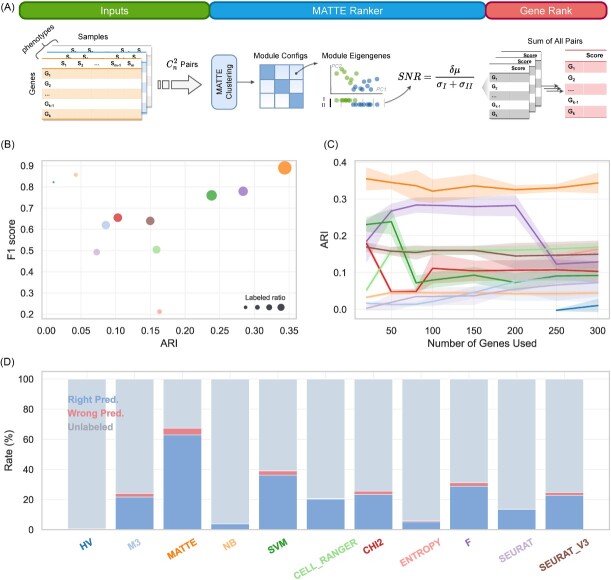
Gene Ranking and results of scRNA-seq data. Bubble color in figure b and line color in figure c indicates the method used, which corresponds to the color of x-axis labels in figure d. Part of abbreviation used the figure: Brennecke’s highly variable method (HV), M3Drop (M3), depth-adjusted negative binomial model (NB), chi-square statistic (CHI2), F-statistic (F). (**A**) Modified Pipeline of MATTE used in gene ranking: each phenotype pairs will be evaluated separately and SNR of each module will be taken as a gene score. Results from each turn will be added as final score. (**B**) Comprehensive performance of methods in this task. (**C**) With the number of marker genes increases, the performance of unsupervised learning task changes. (**D**) Average ratio of prediction of methods.

These simulation experiments may not fully reflect the advantages of MATTE, mainly since the differential expression mode is limited to the generation of simulated data. We further tested MATTE on the single-cell data, as the unique noise of single-cell data is that many low-expressed genes cannot be identified, known as dropouts, because of low sequencing sensitivity. Identifying cell types is necessary for analyzing single-cell data, and current marker-based single-cell annotation tools rely heavily on prior information [34], that is, marker lists. In particular, immune cells can have different cell type annotation depths, resulting in poor performance of current marker-based methods [34, 35]. This section attempts to identify robust cell-type specific markers using modules constructed by MATTE, thereby facilitating the essential annotation of single-cell RNA-seq data. A modified method of MATTE identifies markers in view of the module as shown in Figure 3A. In brief, a gene score is calculated as the sum of the SNRs of the modules to which gene belongs in each phenotype pair.

We applied eleven methods to PUMCBench [36], where seven sequencing technologies are applied to peripheral blood mononuclear cell samples. The 10Xv2 data set was used as the training set to search for critical marker genes in six cell types appearing in all data sets. Eleven methods include MATTE, classical feature selection method (F static, F; Chi-squared, CHI2), differential co-expression method (ENT), differential gene identification method for single-cell data (depth-adjusted negative binomial model, NB; Brennecke’s highly variable gene method [37], HV; M3Drop [38], M3), unsupervised highly variable gene selection techniques (cell ranger [39], Seurat and Seurat v3 [40]) and input variable weight from a support vector machine (SVM) model with probability linear kernel. Each method assigns a score to a gene representing its importance for identifying these cell types. Some methods mentioned previously are not scalable to such large data, such as ECF, and thus are not included.

First, a supervised task is applied to determine whether cells in test datasets can be accurately identified by a classifier trained with a gene list ranked by methods. The corresponding score list (Supplementary Table S1) is applied to the SVM-based classifier with a linear kernel function. Based on the recommendations of a previous study [34], we labeled cells with a prediction probability ˂0.7 as unknown, and calculated the F1 score as an evaluation index in the labeled samples. To rule out the influence of the number of the best-selected genes on our test results, 20–300 genes were selected, and the best results were analyzed. The results show that the classifier trained with MATTE-selected genes (Supplementary Table S2) can identify the greatest number of cells across all (Figure 3D) and achieve the highest F1 score (Figure 3B). Because the expression of genes with low expression has a high variance due to dropouts, some variance-based approaches designated the smallest cells. Too many genes have extremely low *P*-values and are easily mistaken for markers in these techniques. A cell umap [41, 42] atlas of the 10Xv2 dataset also shows the advantage of MATTE (Supplementary Figure S4), in which most cells are labeled and the cell-type location is similar to the ground truth. These results (Supplementary Table S3) showed that MATTE could identify essential genes representing cell types and scored significantly higher on the classification task than other methods.

As the number of selected genes increases, the prediction accuracy of previous classification tasks will also gradually increase because of the low weight of the confounder genes in the SVM classifier. The classification cannot reflect the existence of confounders in the rank. Therefore, we tested it further in an unsupervised task with hierarchical clustering in the Euclidean distance. ARI is used as an evaluating indicator. Then, if the gene list includes more confounder genes, the corresponding ARI will decrease; in contrast, ARI increases when critical genes are included. As a result, there are many confounding genes in the marker list identified by some methods; that is, the ARI decreases with an increase in the number of genes used (Figure 3C). In contrast, the MATTE-sequenced list contained fewer confounding genes, and ARI may even rise as the number of genes increases. In summary, MATTE can extract an ideal gene list for each cell type in an impact of the dropout event and outperform in both supervised and unsupervised tasks over other methods, as shown in the bubble plot (Figure 3B).

### MATTE can achieve precise subtyping of BRCA in a module view

We further intend to demonstrate how MATTE investigates a specific biological problem using BRCA data from the TCGA database. Breast cancer is one of the most common cancers in women, and the detection of subtypes of BRCA has attracted much attention [43–45]. The most commonly used PAM50 subtyping [46] can be used to select an appropriate treatment for patients. What attracts the most research attention is triple-negative breast cancer (TNBC), which lacks therapeutic targets and effective drugs [43, 48], causing challenges in medicine. In recent years, many studies have focused on finding druggable targets and developing drugs available for TNBC [47, 48]. Here, we hope to cluster BRCA patients into several precise subtypes in a module view, understand the potential biological information and promote the development of relevant, targeted drugs.

**Figure 4:**
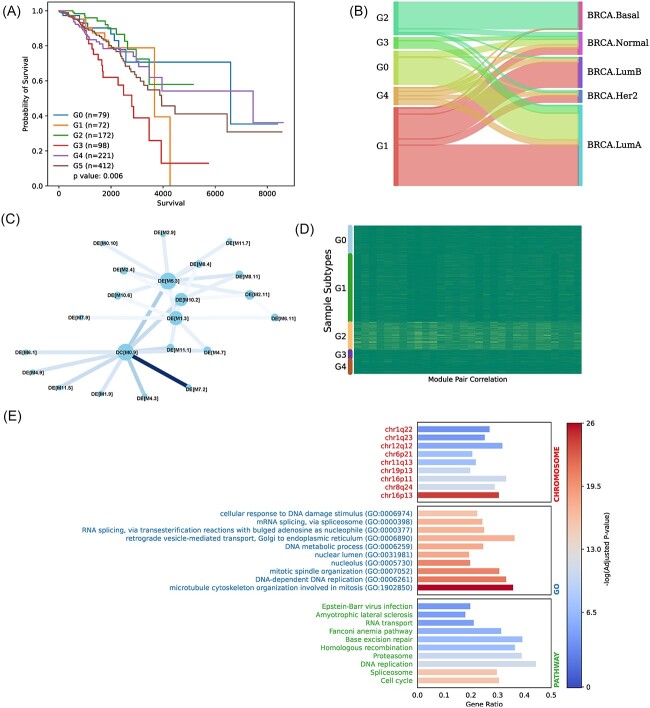
BRCA subtypes detected by MATTE. (**A**) Survival of five BRCA subtypes, getting *P*-value of 0.006 by multivariate log-rank test. Subtypes are labeled ‘G’ followed by a number. (**B**) Sanky plot showing the overlaps of our subtypes with PAM50. (**C**) Core module network specifying G2. To build the network, we first construct a module network based on the ME of each MC in each subtype. The edge weight represents the correlation difference between G2 and other subtypes. The color of edge of the graph represents the difference of G2 and other subtypes. (**D**) Correlation of modules pairs in the figure c. (**E**) Function enrichment analysis of genes in the core module network as figure c draws.

We first performed MATTE-DE and MATTE-DC analysis on cancer versus normal samples from the TCGA database, obtaining modules that characterize BRCA. The following experiments are trying to test if the non-diagonal MCs show differences between BRCA cancer and normal samples. Samples are represented by extracting the difference information contained in the MC genes by PCA, i.e. ME. MEs of the DE and DC processes are combined after filtering the module with an SNR less than a threshold (<0.5) out. Transcriptomics data can be viewed as being embedded in the perspective of MCs as a result of these steps. Then hierarchy clustering with correlation distance was performed to determine BRCA subtypes (Supplementary Table S4). Key features between subtypes are then comprehensively analyzed.

For survival analysis, the log-rank test results showed significant differences between different subtypes (*P*-value = 0.006). We also performed the similar steps in other eight tumor types to identify precise subtypes, including COAD, HNSC, KIRC, KIRP, LIHC, LUAD, LUSC and STAD. The result shows subtypes get a significant difference in survival analysis in all eight tumor types (Supplementary Figure S5, Supplementary Methods). Later, we compared the corresponding clusters with PAM50 and found that G2 highly overlapped with TNBC (Supplementary Figure S6) and PAM50-Basel (Figure 4B). It is therefore important to continue researching G2 typing characteristics from a module perspective. A differential module network was constructed by ME correlation by IIC [25] to significantly distinguish G2 from other subtypes (Figure 4C and D). In the network, nodes present the MCs and edges represents the differential correlation between G2 and other subtypes. The genes in the core network were analyzed by functional enrichment, including chromosome, gene ontology (GO) and pathways (Supplementary Table S5). Regarding the enrichment of a chromosome location, chr16p13 is exceptionally significant. Numerous investigations through literature searches have proven that mutations and aberrant expression in the region are strongly associated with breast cancer [49–51]. One of the genes in this area, PDPK1, phosphorylates and activates a subset of AGC protein kinases that are downstream of PI3K [52], and involved in PI3K/Akt pathways. The chr16p13 region is also related to other cancers, such as prostate cancer [53]. Additionally, GO-enriched items show that the microtubule cytoskeleton involved in mitosis is highly correlated with the development of TNBC, which is consistent with clinical practice and previous research [54, 55]. These findings suggest that MATTE can be utilized to identify differences in the transcriptome and to isolate biologically significant modules for phenotype explanations.

**Figure 5:**
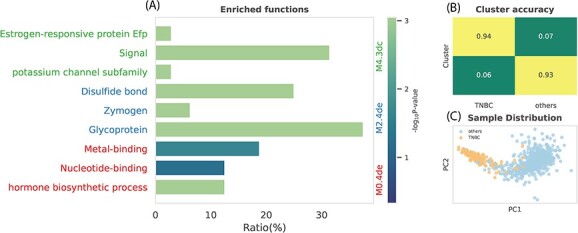
TNBC specific module analysis. (**A**) Enriched function items for three MCs (red: M0.4de; blue: M2.4de; green: M4.3dc). (**B**) Accuracy matrix of clustering label with grand truth. Labels is clustered by K-Means using MEs as samples’ feature. (**C**) Distribution of TNBC samples and non-TNBC samples after PCA dimension reduction.

Furthermore, we turn our attention to the difference between triple-negative and non-triple-negative. A similar analysis is performed except genes in diagonal MCs in the previous analysis are included. Using top three highest SNR MCs’ MEs as sample features, BRCA samples can be clustered by K-means into two groups, roughly equal to TNBC and non-TNBC patients (Figure 5B and C). High cluster accuracy (94% and 93%, respectively) proved that these MCs are differentiated. Functional enrichment was conducted by DAVID [56] to annotate the potential mechanism of TNBC in each MC. Three MCs are related to signal and potassium channels, glycoproteins and hormone biosynthetic processes (Figure 5A). To date, several studies have investigated the relationship of glycoproteins [57–59] as well as potassium channels [60, 61] with tumor growth, metastasis and cell death in TNBC.

## DISCUSSION

We have introduced a novel technique called MATTE for constructing and aligning modules to find differences from transcriptomics data. MATTE shows its robustness and accuracy across a variety of technologies. The framework of MATTE is adaptable and enables the blending of DE and DC analysis as well as potential future extensions to other omics analyses. Through in-depth evaluations and applications of three studies, we have demonstrated the advantages of MATTE.

MATTE relies on relative difference transformation for representing genes from each condition in a denoised matrix. It is conceivable that if noise is generated for the whole, such noise will be eliminated in the comparison between a gene and the others, while those genes that produce differences will make the differences more evident compared with context genes. Module alignment is also beneficial for dealing with noise in transcriptomics data. In our method, the change in genes in the clustering results replaces the specific distance calculation to reduce the impact of random disturbance on the final results. Furthermore, the number of clusters is the scale or resolution of the analysis, which enables flexibility usage to suit all conditions. The cluster number can also be easily decided by cluster quality statistics such as the silhouette score or approaches such as the elbow method. Although there are various methods to eliminate the corresponding noise, it is still valuable to construct methods with the anti-noise ability.

On this issue, module views can provide additional information, according to the theory of causal emergence [62]. Although the perspective of modules has been applied to many studies and methods, aligning modules has not been considered in recent years. We do not try to analyze and align modules that have been detected but identify modules from the raw data. In this way, it is hoped to reduce the error caused by building modules separately and accumulate in the final result. In the future, we hope to introduce network theory and deep learning methods into MATTE. As mentioned above, the expression of a single gene is considered in a broad context, but not all genes are related. Thus, it is attractive to introduce transformer models, attention strategies and graph neural networks into this field.

## CONCLUSIONS

Here, we introduce MATTE, a method for comparing the transcriptomes of various situations and phenotypes. By assuming that genes cooperate, MATTE offers a general solution for creating and aligning denoised modules. It outputs differences in the form of MCs, which could be understood as gene sets and be used to rank genes. Using three studies, we have illustrated the benefits of MATTE and how a module view analysis can provide novel biological insights. In simulations, RDE and RDC have shown their anti-noise ability over other methods. In real single-cell RNA-seq data, MATTE detects better markers used in cell type identification. Finally, we analyzed BRCA transcriptomics data from TCGA to obtain precise subtyping and knowledge of TNBC. In summary, MATTE is a tool for analyzing comparative transcriptomics by creating a discriminative representation and combining clustering with cross-tabulation, which has the advantages of processing noise and explaining the results.

**Key Points**

- We propose a novel transcriptome analysis framework MATTE that aims to align gene modules associated with different phenotypes by modeling phenotype differences as gene location changes.
- Relative differential expression used in this study is able to process both differential expression and differential co-expression patterns and is expected to represent genes better as noise is eliminated by comparing to the background gene expression.
- MATTE constructs and aligns modules simultaneously by a cross-tabulation method that does not depend on the number of genes, but identifies the component differences of each module. Thus, MATTE can interpret both conserved and differentiated modules.
- The validation results show the ability of MATTE in identifying representative marker genes under random noise, batch-effect-like noise and single-cell RNA-seq dropout noise. By analyzing TCGA-BRCA data, MATTE is also able to interpret disease in a module view.

## Supplementary Material

Additional_file_1_R2_bbad207

Additional_file_2_bbad207

## Data Availability

The BRCA transcriptomics data are extracted from TCGA project (https://portal.gdc.cancer.gov/). Preprocessed pan-cancer transcriptomics data are extracted from Xenahub (https://tcga.xenahubs.net). The single-cell RNA-seq datasets were first generated by Ding *et al.* [36] and further reorganized by Abdelaal *et al.* [34, 63].

## Author Biographies

**Guoxing Cai** is currently a PhD candidate at the Innovation Institute for Artificial Intelligence in Medicine and Zhejiang Provincial Key Laboratory of Anti-Cancer Drug Research, College of Pharmaceutical Sciences, Zhejiang University. His main research interests include the development of bioinformatics tools and cancer transcriptome analysis.

**Wenyi Zhao** is a postdoctoral associate at the Innovation Institute for Artificial Intelligence in Medicine and Zhejiang Provincial Key Laboratory of Anti-Cancer Drug Research and the Innovation Institute for Artificial Intelligence in Medicine, College of Pharmaceutical Sciences, Zhejiang University. Her main research interests include the development of bioinformatics tools and databases and cancer genomics.

**Zhan Zhou** is an associate professor at the Innovation Institute for Artificial Intelligence in Medicine and Zhejiang Provincial Key Laboratory of Anti-Cancer Drug Research, College of Pharmaceutical Sciences, Zhejiang University; an adjunct professor at the Fourth Affiliated Hospital, Zhejiang University School of Medicine. His main research interests include the development of bioinformatics tools and databases and cancer genomics.

**Xun Gu** is a professor at the Department of Genetics, Development and Cell Biology, Iowa State University. His main research interests include evolutionary genomics and molecular evolution.
