# Supplementary material for: MATTE: a pipeline of transcriptome module alignment for anti-noise phenotype-gene-related analysis
Source: Brief Bioinform. 2023 Jun 3;24(4):bbad207. doi: 10.1093/bib/bbad207 (PMC10359084; doi:10.1093/bib/bbad207)
Supplement: Additional_file_1_R2_bbad207 [file additional_file_1_r2_bbad207.pdf]

Additional file 1: Supplementary information for

MATTE: a pipeline of transcriptome module alignment for anti-noise phenotype-gene-related analysis

Guoxin Cai[1], Wenyi Zhao[1], Zhan Zhou[1,3,*], Xun Gu[2,*]

[*] Corresponding authors: Zhan Zhou, zhanzhou@zju.edu.cn; Xun Gu, xgu@iastate.edu.

Supplementary Methods

Notation

We use small cases to represent a vector, i.e., x and large cases to represent a matrix, i.e., X. $\langle x \rangle = 1/m \sum_i^m x_i$ and $\sigma(x)$ represent mean value and standard deviation of a vector x respectively. $[X, Y]$ represents the concatenate of two matrixes X and Y. pi and gi represents different phenotypes and genes. $\{gi; condition\}$ is a gene set that meets the condition.

Ability of anti-noise

In this section, we aim to discuss the reason and hypothesis why relative difference remove the noise. Consider that gene expression X is influenced by three factors: phenotype effects $\hat{X}$, individual variability ϵ_s, and batch effects ϵ_b, where $\epsilon_s \sim N(0, \sigma_{\epsilon s})$ and $\epsilon_b \sim N(\mu_b, \sigma_{\epsilon b})$.

$$X = \hat{X} + \epsilon_s + \epsilon_b \tag{1}$$

Calculate the mean value of gene expression eliminate the individual variability as $\sum_s \epsilon_s \to 0$.

$$\langle X \rangle = \langle \hat{X} \rangle + \langle \epsilon_b \rangle + \langle \epsilon_s \rangle = \langle \hat{X} \rangle + \langle \epsilon_b \rangle \tag{2}$$

As batch effect between two phenotypes can be different, the noise must be eliminated before any calculation between two source data. By considering $\epsilon_b^i - \epsilon_b^j \to 0$, we get RDE definition.

$$RDE \doteq \left| \langle X_i \rangle - \langle X_j \rangle \right| = \left| \langle \hat{X}_i \rangle - \langle \hat{X}_j \rangle \right| \tag{3}$$

Inter-individual correlation

In this section, we will show why how inter-individual correlation represents the gene co-expression in a view of individual. For any two gene expressions x and y, let

$$C_i^{xy} = \frac{(x_i - \langle x \rangle)(y_i - \langle y \rangle)}{\sigma_x \sigma_y} \tag{4}$$

where σ is the standard variance of a gene, $\bar{x}$ and $\bar{y}$ are the mean of the expression. Then the mean of C is equal to the Pearson's correlation coefficient ρ.

$$\langle C^{xy} \rangle = \frac{1/N \times \sum_{i=1}^{N}(x_i - \langle x \rangle)\,(y_i - \langle y \rangle)}{\sigma_x \sigma_y} \equiv \rho_{x,y} \tag{5}$$

Thus, C can be seen as the sample resolution co-expression strength of the two genes. In this way, a similar strategy can be used to explore the gene pair with co-expression difference between two phenotypes.

Briefings to the compared methods

Differential Co-expression. Expected conditional F statistic (ECF) [1] calculates the F statics under the expected condition. Python implementation refers to the R package cosine [2]. The following three methods' python implementation refers to R package dcanr [3]. Z-score[4] converts the PCC of gene pairs into statistics of gene triplets. Entropy [5] of PCC can be calculated based on probabilistic graphical models. DiffCoEX [6] constructs a scale-free network as WGCNA does and uses the topological overlap to calculate differential co-expression.

Feature ranking methods. ANOVA F-value is based on the sum of squares and the ratio of intra-and inter-group deviations in different label groups. In this study, the features are ranked by the value of the F statistic. The chi-square value measures the dependency and independence between data and labels. Mutual information (MI) measures the variables' dependence which equal to zero when two random variables are independent, while higher values indicate greater reliance. Signal-to-noise ratio (SNR) is a commonly used metrics for the significance of observation. In this study, for SNR of genes between two groups, signal is defined as the absolute subtraction of mean values in two groups, and noise is defined as sum of the standard deviations in two groups.

DE methods designed for scRNA. M3drop [7] is an R package for single-cell expression data's DE analysis. Three differential expression methods were used in this study. M3Drop: Under the null hypothesis, it is considered that the dropout ratio and average gene expression follow the Michaelis–Menten equation. The negative binomial model (NB) models each observation as a negative binomial distribution. Brennecke's highly variable gene method (HVG) looks for genes with significant changes in gene expression. It is considered that there is a linear relationship between the square of the coefficient of variation and the average amount of expression.

Other methods. Three unsupervised methods are based on a hypothesis that highly variant genes are tend to be important. While implementation details differ as follows. Seurat v3 HVG [8] ranking is based on a variance stabilizing transformation. Cell ranger [9] and Seurat HVG [8] ranking the dispersions of each bin which is separated by the mean value of genes. Implementation of above three unsupervised methods are based on scanpy python package [10]. Model based method extracts the weight of each gene from a SVM model with linear kernel.

Pan-cancer analysis

We have collected the pan-cancer transcriptomics data and clinical information at Xenahub (https://tcga.xenahubs.net, version 2016-12-29), including colon adenocarcinoma (COAD, 449 cancer samples and 43 normal samples), head and neck squamous cell carcinoma (HNSC, 520 cancer samples and 46 normal samples), Kidney renal clear cell carcinoma (KIRC, 533 cancer samples and 73 normal samples), Kidney renal papillary cell carcinoma (KIRP, 290 cancer samples and 33 normal samples), Liver hepatocellular carcinoma (LIHC, 371 cancer samples and 53 normal samples), Lung adenocarcinoma (LUAD, 515 cancer samples and 61 normal samples), Lung squamous cell carcinoma (LUSC, 501 cancer samples and 52 normal samples) and Stomach adenocarcinoma (STAD, 415 cancer samples and 35 normal samples).

For pan-cancer analysis, MATTE is first performed to obtain MCs of each cancer type that characterize the cancer (compared to normal samples). Then, each sample is represented by the eigengene of MCs whose SNR is above 0.5. For subtyping, agglomerative clustering is performed based on the correlation distance. For the classification of cancer and normal samples, logistic regression is used.

Function enrichment analysis

Function enrichment analysis is performed in the DAVID web server [11]. Function annotations includes gene ontology, KEGG pathway and cytoband information.

References

1. Lai Y, Wu B, Chen L, et al. A statistical method for identifying differential gene-gene co-expression patterns. Bioinformatics 2004; 20:3146–3155
2. Ma H, Schadt EE, Kaplan LM, et al. COSINE: COndition-SpecIfic sub-NEtwork identification using a global optimization method. Bioinformatics 2011; 27:1290–1298
3. Bhuva DD, Cursons J, Smyth GK, et al. Differential co-expression-based detection of conditional relationships in transcriptional data: comparative analysis and application to breast cancer. Genome Biology 2019; 20:236
4. Zhang J, Ji Y, Zhang L. Extracting three-way gene interactions from microarray data. Bioinformatics 2007; 23:2903–2909
5. Segal E, Shapira M, Regev A, et al. Module networks: identifying regulatory modules and their condition-specific regulators from gene expression data. Nature Genetics 2003; 34:166–176
6. Tesson BM, Breitling R, Jansen RC. DiffCoEx: a simple and sensitive method to find differentially coexpressed gene modules. BMC Bioinformatics 2010; 11:497
7. Andrews TS, Hemberg M. M3Drop: dropout-based feature selection for scRNASeq. Bioinformatics 2019; 35:2865–2867
8. Satija R, Farrell JA, Gennert D, et al. Spatial reconstruction of single-cell gene expression data. Nat Biotechnol 2015; 33:495–502
9. Zheng GXY, Terry JM, Belgrader P, et al. Massively parallel digital transcriptional profiling of single cells. Nat Commun 2017; 8:14049
10. Wolf FA, Angerer P, Theis FJ. SCANPY: large-scale single-cell gene expression data analysis. Genome Biology 2018; 19:15
11. Sherman BT, Hao M, Qiu J, et al. DAVID: a web server for functional enrichment analysis and functional annotation of gene lists (2021 update). Nucleic Acids Research 2022; gkac194

Supplementary Figures

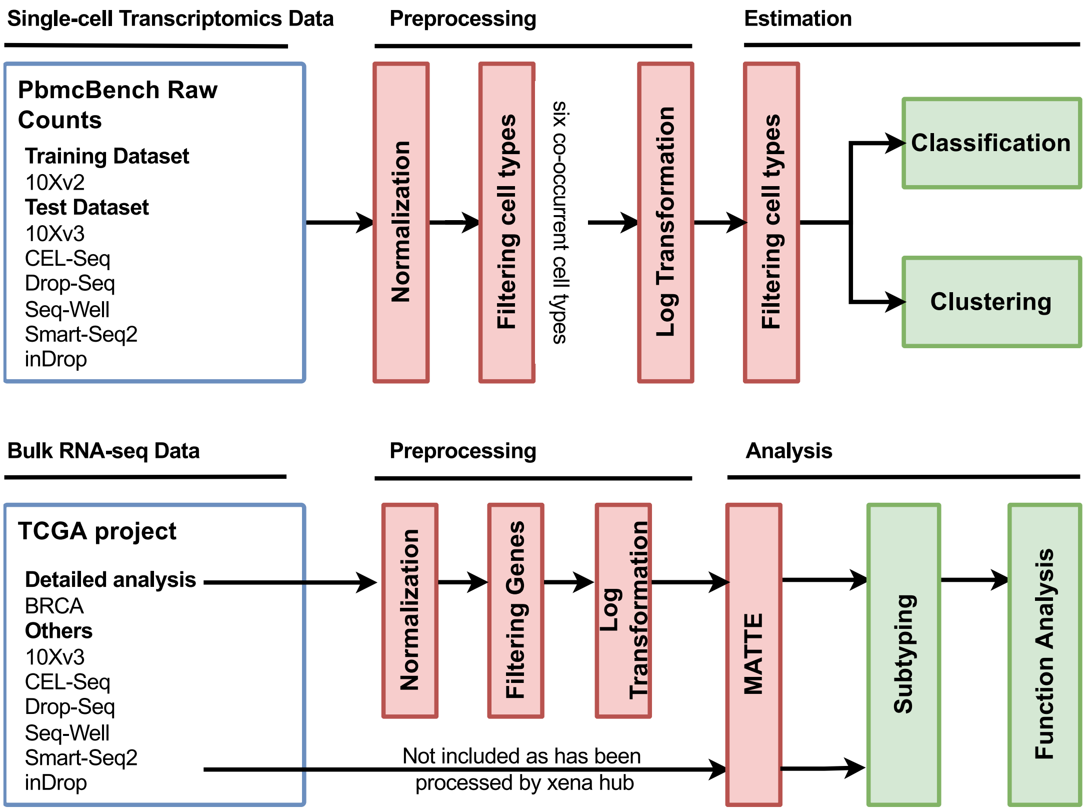

Figure S1. Summary of data processing, estimation and analysis in this study. For single-cell transcriptomics data, preprocessing including: (1) Normalization (2) Filtering cell types: only six cell types that appeared in all datasets are used. (3) Log-transformation. For BRCA transcriptomics data, which is downloaded from TCGA project, preprocessing including: Normalization, Filtering Genes and Log-transformation. For other bulk RNA-seq data, which is downloaded from XenaHub, no preprocessing is needed as has been done by the Xenahub.

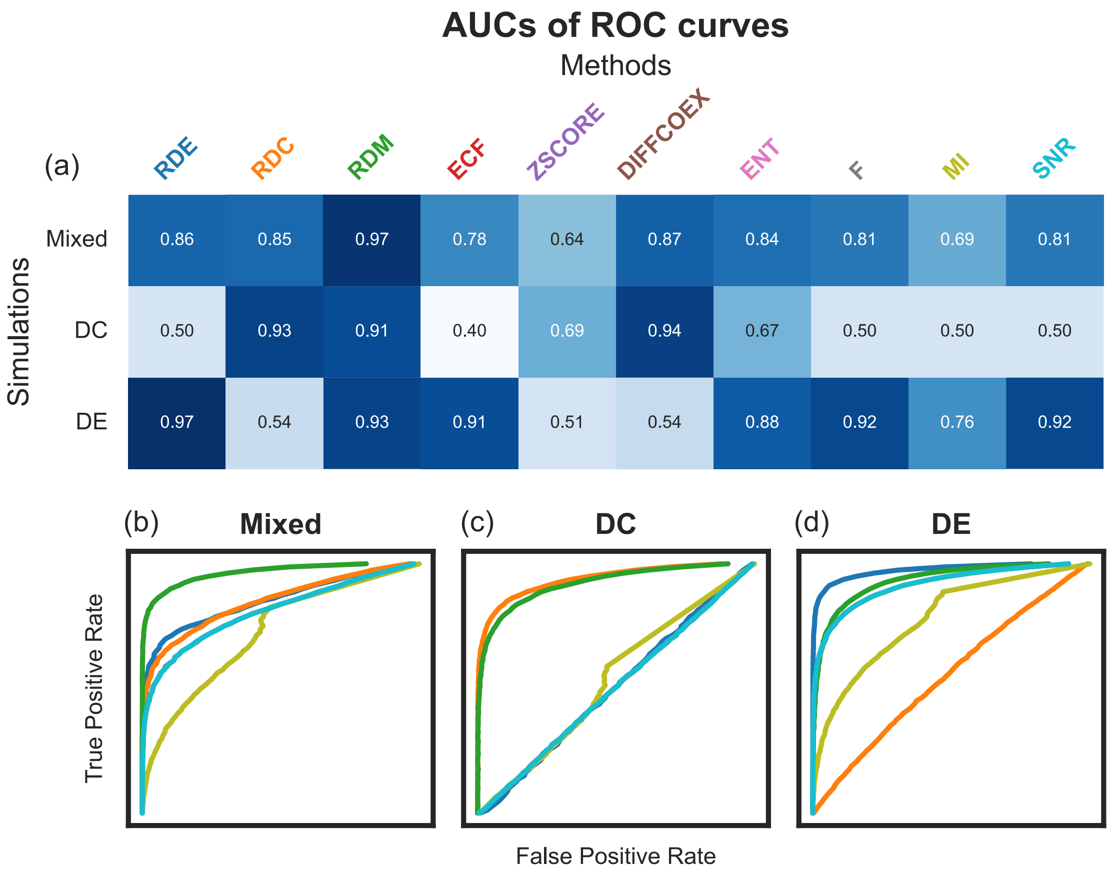

Figure S2. ROC curves and its AUC in the simulation. Color of heatmap index is corresponds to ROC curve color. (a) AUC of ROC of each method in each simulation data (number of iterations equals to 100). (b-d) ROC curve under each simulation.

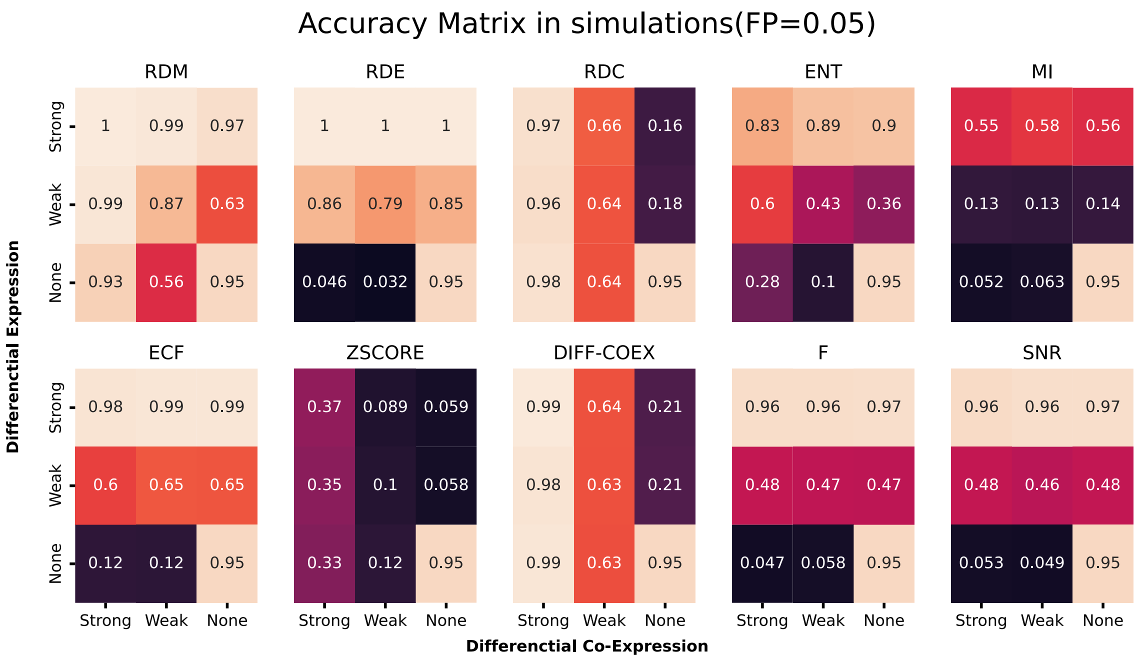

Figure S3. Accuracy of each component in simulations (mix patterns). For easy comparison, threshold is set for each method when false positive (FP) is 0.05.

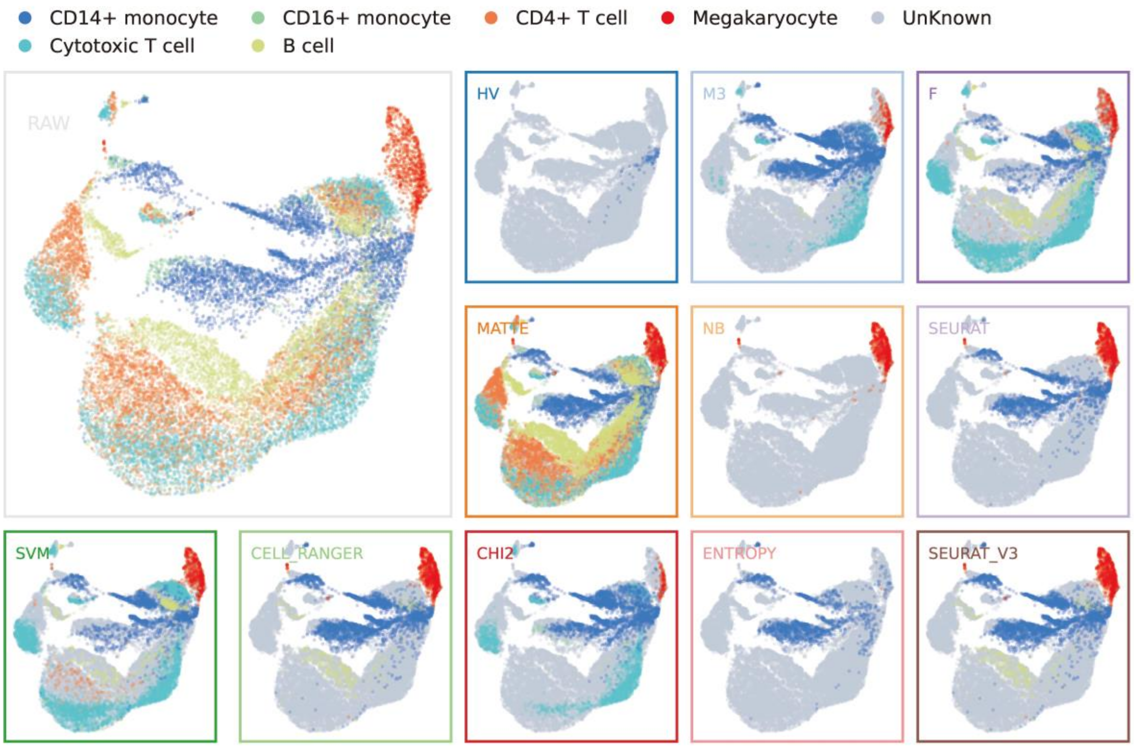

Figure S4. U-map visualization of predictions of different methods. Each color refers to a cell type, while grey presents unknown cell types (probability of prediction is lower than 0.7).

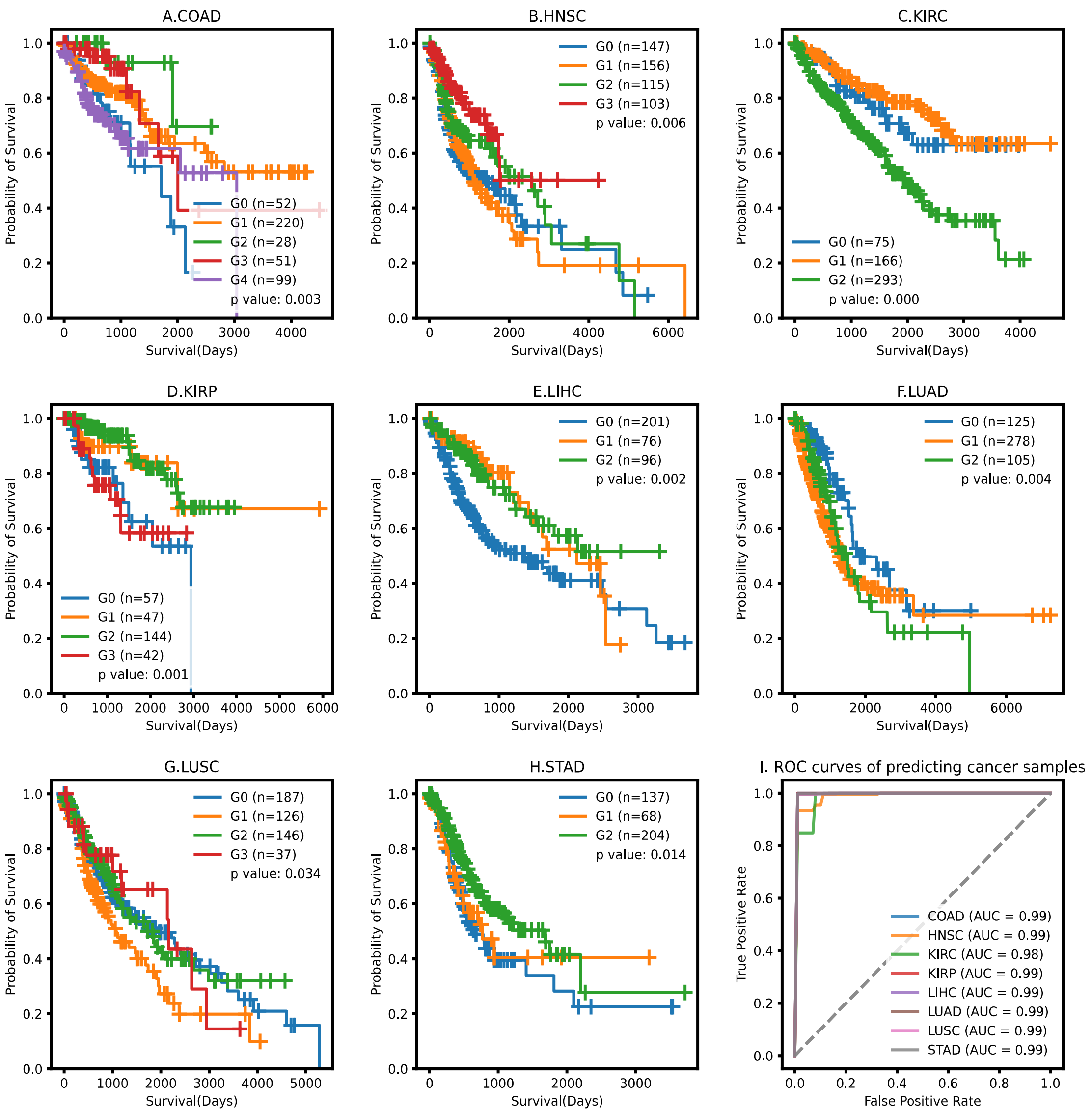

Figure S5. MATTE performs in eight tumor types. (A-H) Survival analysis of subtyping. Title represents the TCGA abbreviation of cancers. Log rank test is performed to get the p value. The subtyping is based on a similar strategy of BRCA subtyping. In brief, representation based on MCs comparing to the normal samples is used to agglomerative clustering. (I) ROC curves of predicting cancer samples. Logistic regression models are used to predict sample labels in the MC-based representation. COAD: colon adenocarcinoma, HNSC: head and neck squamous cell carcinoma, KIRC: Kidney renal clear cell carcinoma, KIRP: Kidney renal papillary cell carcinoma, LIHC: Liver hepatocellular carcinoma, LUAD: Lung adenocarcinoma, LUSC: Lung squamous cell carcinoma and STAD: Stomach adenocarcinoma.

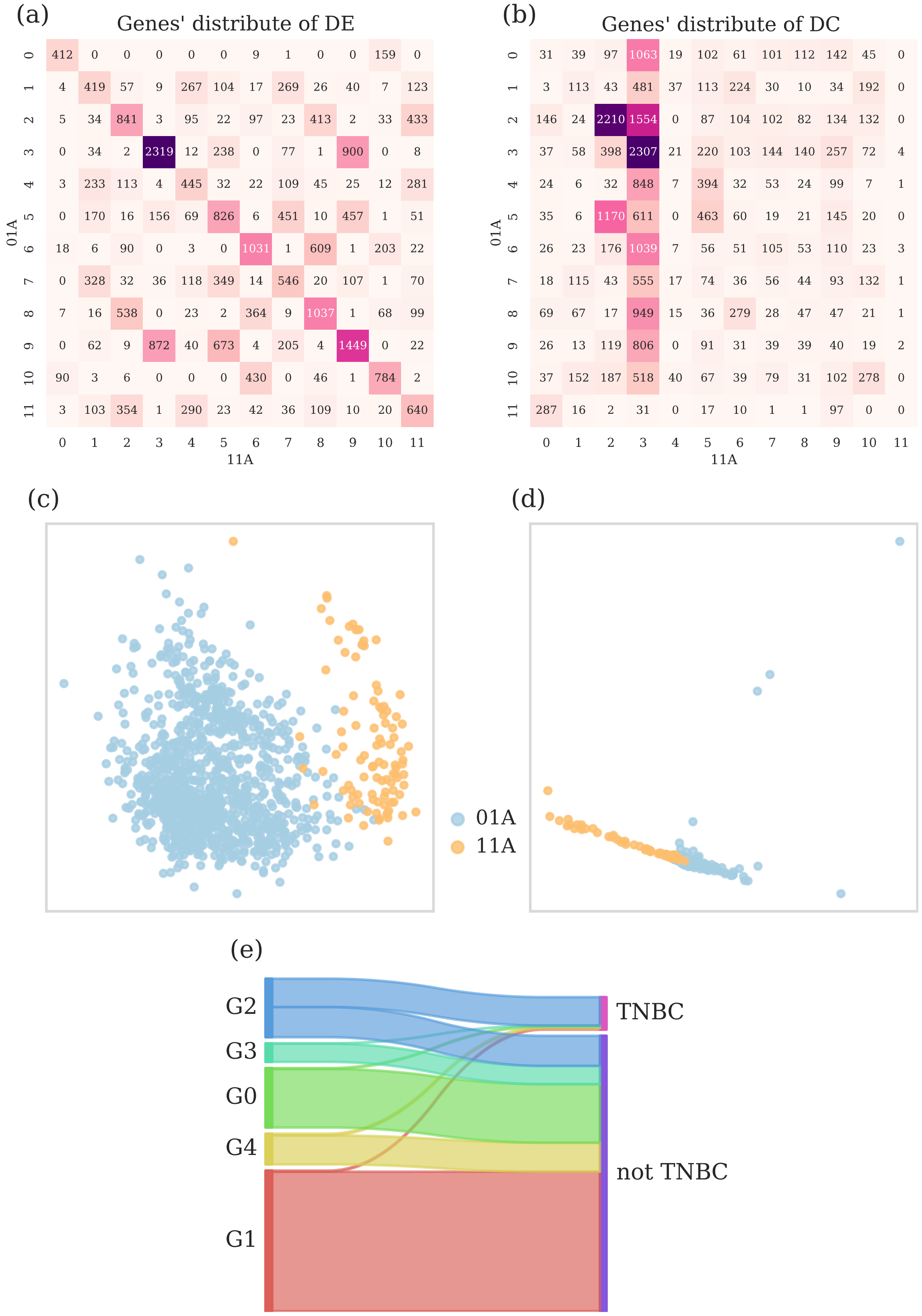
(a)
Genes' distribute of DE
01A
11A
0 1 2 3 4 5 6 7 8 9 10 11
0: 412 0 0 0 0 0 9 1 0 0 159 0
1: 4 419 57 9 267 104 17 269 26 40 7 123
2: 5 34 841 3 95 22 97 23 413 2 33 433
3: 0 34 2 2319 12 238 0 77 1 900 0 8
4: 3 233 113 4 445 32 22 109 45 25 12 281
5: 0 170 16 156 69 826 6 451 10 457 1 51
6: 18 6 90 0 3 0 1031 1 609 1 203 22
7: 0 328 32 36 118 349 14 546 20 107 1 70
8: 7 16 538 0 23 2 364 9 1037 1 68 99
9: 0 62 9 872 40 673 4 205 4 1449 0 22
10: 90 3 6 0 0 0 430 0 46 1 784 2
11: 3 103 354 1 290 23 42 36 109 10 20 640
(b)
Genes' distribute of DC
01A
11A
0 1 2 3 4 5 6 7 8 9 10 11
0: 31 39 97 1063 19 102 61 101 112 142 45 0
1: 3 113 43 481 37 113 224 30 10 34 192 0
2: 146 24 2210 1554 0 87 104 102 82 134 132 0
3: 37 58 398 2307 21 220 103 144 140 257 72 4
4: 24 6 32 848 7 394 32 53 24 99 7 1
5: 35 6 1170 611 0 463 60 19 21 145 20 0
6: 26 23 176 1039 7 56 51 105 53 110 23 3
7: 18 115 43 555 17 74 36 56 44 93 132 1
8: 69 67 17 949 15 36 279 28 47 47 21 1
9: 26 13 119 806 0 91 31 39 39 40 19 2
10: 37 152 187 518 40 67 39 79 31 102 278 0
11: 287 16 2 31 0 17 10 1 1 97 0 0
(c)
(d)
01A
11A
(e)
G2
G3
G0
G4
G1
TNBC
not TNBC

Figure S6. (a) Gene distribution result of DE analysis. Rows and columns represent input phenotypes or conditions. The number of the block represents the number of gene of this MC. (b) Gene distribution result of DC analysis. (c-d) Sample distribution for DE and DC respectively after PCA dimension reduction of non-diagonal MCs' ME. (e) Sanky plot showing the overlaps of our subtypes with TNBC. 11A: TCGA sample ID ends with 11A, represents normal samples. 01A: TCGA sample ID ends with 01A, represents tumor samples.
